# Menin Links the Stress Response to Genome Stability in *Drosophila melanogaster*

**DOI:** 10.1371/journal.pone.0014049

**Published:** 2010-11-18

**Authors:** Maria Papaconstantinou, Alicia N. Pepper, Ying Wu, Dahlia Kasimer, Tim Westwood, Ana Regina Campos, Pierre-André Bédard

**Affiliations:** 1 Department of Biology, McMaster University, Hamilton, Ontario, Canada; 2 Department of Cell and Systems Biology and Canadian Drosophila Microarray Centre, University of Toronto, Mississauga, Ontario, Canada; University of Texas MD Anderson Cancer Center, United States of America

## Abstract

**Background:**

The multiple endocrine neoplasia type I gene functions as a tumor suppressor gene in humans and mouse models. In *Drosophila melanogaster*, mutants of the menin gene (*Mnn1*) are hypersensitive to mutagens or gamma irradiation and have profound defects in the response to several stresses including heat shock, hypoxia, hyperosmolarity and oxidative stress. However, it is not known if the function of menin in the stress response contributes to genome stability. The objective of this study was to examine the role of menin in the control of the stress response and genome stability.

**Methodology/Principal Findings:**

Using a test of loss-of-heterozygosity, we show that *Drosophila* strains lacking a functional *Mnn1* gene or expressing a *Mnn1* dsRNA display increased genome instability in response to non-lethal heat shock or hypoxia treatments. This is also true for strains lacking all *Hsp70* genes, implying that a precise control of the stress response is required for genome stability. While menin is required for *Hsp70* expression, the results of epistatic studies indicate that the increase in genome instability observed in *Mnn1* lack-of-function mutants cannot be accounted for by mis-expression of *Hsp70*. Therefore, menin may promote genome stability by controlling the expression of other stress-responsive genes. In agreement with this notion, gene profiling reveals that *Mnn1* is required for sustained expression of all heat shock protein genes but is dispensable for early induction of the heat shock response.

**Conclusions/Significance:**

Mutants of the *Mnn1* gene are hypersensitive to several stresses and display increased genome instability when subjected to conditions, such as heat shock, generally regarded as non-genotoxic. In this report, we describe a role for menin as a global regulator of heat shock gene expression and critical factor in the maintenance of genome integrity. Therefore, menin links the stress response to the control of genome stability in *Drosophila melanogaster*.

## Introduction

The multiple endocrine neoplasia type 1 tumor suppressor gene (*MEN1*) has been implicated in the control of apoptosis, DNA repair or replication, and gene expression [1]. Menin, the protein encoded by *MEN1*, functions as a transcriptional regulator by interacting with several transcription factors and chromatin-modifying complexes such as mSin3A/histone deacetylases and histone methyltransferases of the Trithorax/MLL (Mixed Lineage Leukemia) family [2], [3], [4], [5], [6], [7], [8]. Leukemogenesis resulting from chromosomal translocations of the MLL1 gene is characterized by the aberrant expression of *Hox* genes, a process dependent on the interaction of the MLL fusion protein with menin [9]. Therefore, menin also functions as an oncogenic co-factor. In contrast, little is known about the mechanism(s) by which menin exerts its tumor suppressor function. Complexes of menin with the replication factor RPA2 and DNA repair protein FANCD2 have been described but a role for menin in DNA replication or repair remains poorly understood [10], [11], [12].

Chromosomal abnormalities have been observed in peripheral leukocytes of patients affected by the MEN1 syndrome, suggesting a role for menin in the control of genome stability [13], [14], [15]. Using *Drosophila melanogaster* as a model, Bale and co-investigators described a function for menin in a G1-S checkpoint following treatment of larvae with ionizing radiation (IR). This function appeared to be mediated by the interaction of menin with the Forkhead family member CHES1 (FOXN3) as over-expression of CHES1 was sufficient to re-establish the G1-S checkpoint and inhibition of DNA synthesis in *Mnn1* deletion mutants [5]. These mutants were also prone to single base pair deletions when treated with a DNA crosslinking agent, suggesting that menin promotes genome stability by several mechanisms [12].

Our group uncovered a novel function for menin in the stress response in *Drosophila*. Deletion mutants of the *Mnn1* gene develop normally but are hypersensitive to several stresses including heat shock, hypoxia, hyperosmolarity and oxidative stress [16]. Defects in the expression of two heat shock responsive genes, namely *Hsp70* and *Hsp23*, were observed in embryos of *Mnn1* nullizygous flies while the over-expression of menin impaired the down-regulation of these genes during recovery at the normal temperature. In this report, we examine the role of menin in the control of the stress response and genome stability. We show that menin functions as a global regulator of the stress response. Using a test for loss-of-heterozygosity of the *multiple wing hairs* (*mwh*) gene, we demonstrate that *Mnn1* nullizygous flies are characterized by increased genome instability in response to heat shock and hypoxia. A proper response to stress, including conditions generally not regarded as genotoxic (heat shock), is therefore required not only for the immediate survival of the organism but also to ensure genome stability, a process depending on menin.

## Results

### Role of menin in genome stability

**Figure 1:**
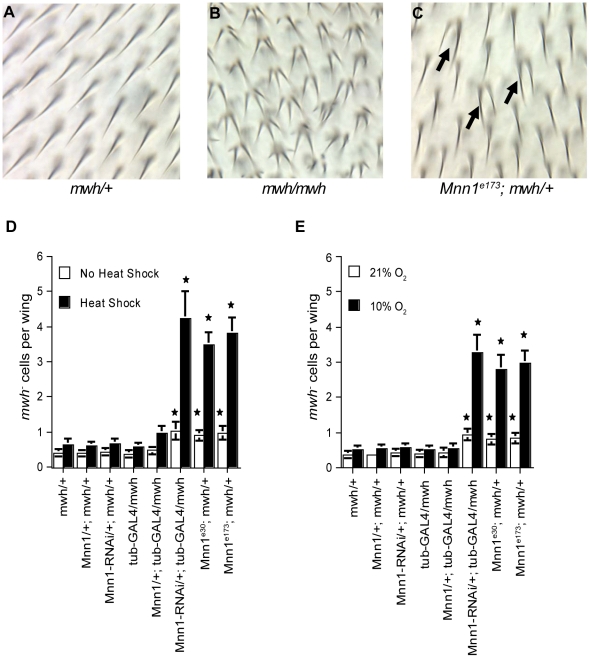
Elevated rates of spontaneous and stress-induced loss-of-heterozygosity in *Mnn1* RNA*i* and deletion mutants. A loss-of-heterozygosity (LOH) assay based on mutations in the *multiple wing hairs* (*mwh*) gene was used to assess genomic instability in *Mnn1* transgenic strains and deletion mutants. (**A**) A fly heterozygous for *mwh* has one wing hair per cell. (**B**) A fly homozygous for *mwh* has two or more hairs per cell. (**C**) LOH is elevated in *Mnn1^e173^* deletion mutant flies. Individual cells that have lost the wild-type *mwh* gene are observed within a field of heterozygous cells (arrows in **C**). Panels **A** & **C** show individuals that have received short, repeated heat shock treatments. (**D&E**) In the absence of stress, the incidence of *mwh^-^* cells is increased significantly in *Mnn1* deletion mutants (*Mnn1^e30^;mwh/+* and *Mnn1^e173^;mwh/+*) and flies expressing the UAS construct for *Mnn1* RNAi under the control of tubulin-GAL4 (*Mnn1-RNAi/+; tub-GAL4/mwh*) (p<0.05). These elevated rates of LOH are significantly enhanced by short (20 min), repeated heat shock treatments (**D**) and by short (1 hr), repeated hypoxia treatments (**E**). *Mnn1*/+; *tub*-*GAL4*/*mwh* refers to a strain harboring a UAS construct for menin over-expression; this strain does not show a significant difference in the frequency of LOH. The average number of *mwh^-^* cells per wing is indicated. Error bars represent standard errors of the mean (SEM). Stars denote significant differences compared to wild-type controls (*mwh*/+) of the same condition, p<0.05.

The *MEN1* gene functions as a tumor suppressor gene in humans and in mouse models [17], [18], [19], [20]. In *Drosophila*, mutants carrying a deletion of the *Mnn1* gene are hypersensitive to several mutagens or gamma irradiation and display profound defects in the stress response [5], [16], [21]. However, it is not known if the function of menin in the stress response contributes to genome stability. To address this question, we quantified the loss-of-heterozygosity (LOH) at the *multiple wing hairs* (*mwh*) locus in adults that were subjected to heat shock or hypoxia during embryonic and larval stages. In precursor wing cells, loss-of-function at this locus generates cells with multiple hairs in the adult (Fig. 1A–C). Control and *Mnn1* null embryos and larvae heterozygous for the *mwh^1^* mutation were subjected to repeated but short 20 min heat shock treatments at 37°C. These conditions of heat shock caused similar levels of lethality (5–8%) in control and *Mnn1* mutants (Supporting Information Table S1). The repeated heat shock treatment had little effect on genome stability in flies with a functional *Mnn1* gene (Fig. 1D). In contrast, we observed a significant increase in *mwh* LOH in *Mnn1* deletion mutants (*Mnn1^e30^*, *Mnn1^e173^*) or flies expressing a dsRNA for *Mnn1* in response to heat shock (Fig. 1D). The same was true for embryos and larvae subjected to sub-lethal conditions of hypoxia (Fig. 1E). Thus, we conclude that *Mnn1* gene function is required for the maintenance of genome stability in response to stress.

### Role of Hsp70 in genome stability

**Figure 2:**
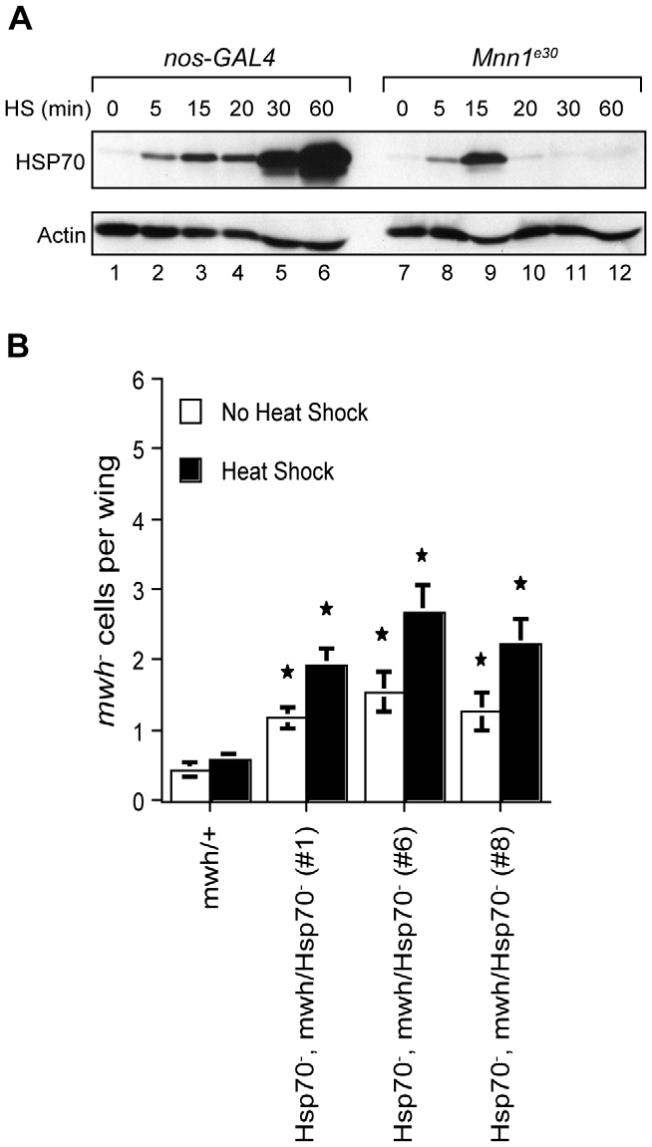
Loss of *Hsp70* leads to increased genomic instability after heat stress. **A**) Western blotting analysis of HSP70 expression in control (*nos*-*GAL4*) and *Mnn1^e30^* deletion mutant embryos. The expression of HSP70 is normal in the first 15 min of the heat shock response but is no longer detectable after a 20 min heat shock treatment in the *Mnn1^e30^* deletion mutant. (**B**) The *mwh* assay was used to measure genomic instability in post-mitotic cells of *Hsp70^-^* mutants. Under normal lab conditions, the incidence of *mwh^-^* cells is significantly increased about two- to three-fold in *Hsp70^-^* deletion mutants in comparison with the controls (*mwh*/+) (p<0.05). These elevated rates of LOH are significantly augmented by short (20 min), chronic heat shock treatments (p<0.05). Both *Hsp70* and *mwh* genes are on the third chromosome, and three independent recombinant lines (#1, #6 and #8) homozygous for *Hsp70^-^* and heterozygous for *mwh* were tested. The average number of *mwh^-^* cells per wing is indicated. Error bars represent SEM. Stars denote significant differences compared to controls (*mwh*/+) for the same condition, p<0.05.

In the absence of *Mnn1* function, *Drosophila* embryos express HSP70 in the first 15 min of heat shock but are unable to maintain this expression for longer periods [16]. Western blotting analysis indicated that embryos with a deletion of the *Mnn1* gene do not express HSP70 after 20 min of heat shock at 37°C, that is, in conditions used to assess the role of menin in the control of genome stability (Fig. 2A). This observation raised the possibility that the lack of proper HSP70 expression is sufficient to cause genome instability. To address this question, we took advantage of deletion mutants of the *Hsp70* loci generated by homologous recombination [22]. The absence of *Hsp70* expression results in increased lethality in response to severe heat shock conditions (39°C) but is tolerated in the heat stress conditions used in our experiments [[23] Supporting Information Table S1 and S2]. Increased *mwh* LOH was observed in *Hsp70* null flies subjected to the repeated heat shock treatment at 37°C (Fig. 2B). A modest but significant increase was also observed in the absence of heat shock, suggesting that proper expression of *Hsp70* is important for the maintenance of genome stability.

**Figure 3:**
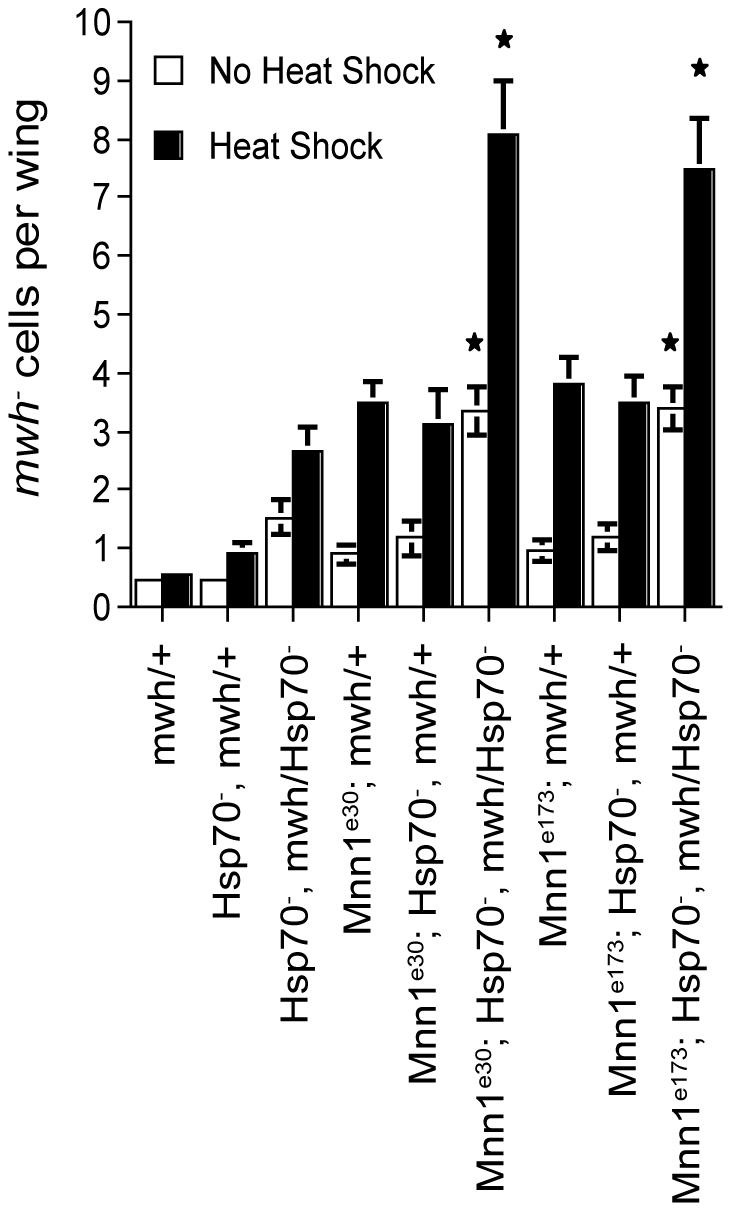
LOH in *Mnn1* deletion mutants is not attributable to the disruption of *Hsp70* expression. The *mwh* assay was used to assess genomic instability in *Mnn1* deletion mutants (*Mnn1* ^e30^ and *Mnn1* ^e173^) harboring half of the *Hsp70* gene copy number or none. Both in the absence of stress or under short, repeated heat shock conditions, a reduction of the *Hsp70* gene complement by half (*Mnn1^e30^*; *Hsp70* ^-^, *mwh*/+ and *Mnn1^e173^*; *Hsp70* ^-^, *mwh*/+), did not result in a significant increase in the incidence of *mwh^-^* cells and did not augment the LOH observed in *Mnn1* null flies (*Mnn1^e30^*; *mwh*/+ and *Mnn1^e173^*; *mwh*/+) (p>0.05). In contrast, under both normal lab and heat shock conditions, the complete loss of *Hsp70* genes (*Mnn1^e30^*; *Hsp70* ^-^, *mwh*/*Hsp70* ^-^ and *Mnn1^e173^*; *Hsp70* ^-^, *mwh*/*Hsp70* ^-^) significantly enhanced the LOH in *Mnn1* deletion mutants (p<0.05). The average number of *mwh^-^* cells per wing is indicated. Error bars represent SEM. Stars denote significant differences compared to *Mnn1* null flies (*Mnn1^e30^*; *mwh*/+ or *Mnn1^e173^*; *mwh*/+), p<0.05.

The genome instability observed in the absence of menin may be caused by the mis-expression of *Hsp70* or disruption of the expression of other genes. To characterize the role of *Hsp70* in the function of menin, LOH at the *mwh* locus was examined in *Mnn1* deletion mutants harboring half of the *Hsp70* gene copy number or none (Fig. 3). The *Drosophila* genome contains six highly similar *Hsp70* genes located at two separate loci. In the presence of menin, a reduction of the *Hsp70* gene complement by half did not result in a significant increase in *mwh* LOH. Likewise, a 50% reduction in *Hsp70* gene copy number did not enhance the incidence of *mwh* LOH observed in *Mnn1* deletion mutants under heat shock conditions. In contrast, the complete absence of *Hsp70* genes resulted in a marked increase in *mwh* LOH in *Mnn1* null flies. This level of LOH was equivalent to the combined *mwh* LOH observed in the absence of *Hsp70* and *Mnn1* gene functions (Fig. 1, 2, 3). This was true at the normal temperature and under heat shock conditions. Therefore, the incidence of LOH observed in *Mnn1* deletion mutants was not the result of the disruption of *Hsp70* expression, suggesting that proper regulation of other genes controlled by menin is required for the maintenance of genome integrity.

### Role of *Hsp70* in the lethality caused by *Mnn1* over-expression

**Figure 4:**
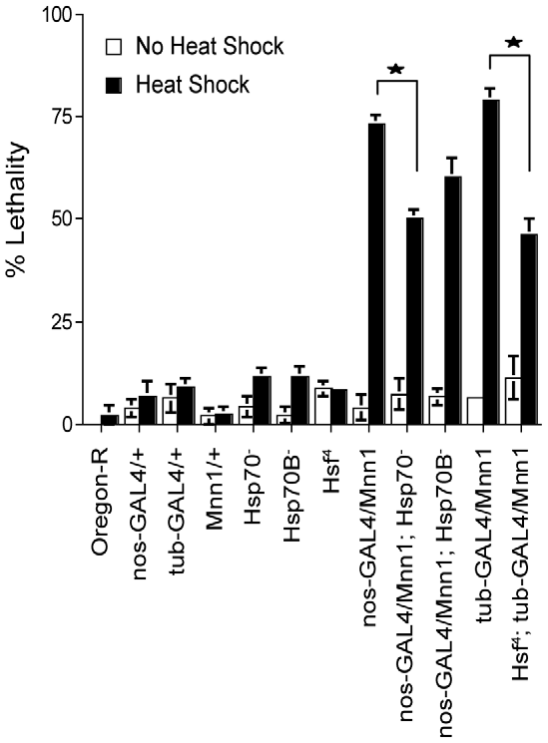
The lethality observed in transgenic strains over-expressing menin is partially due to aberrant *Hsp70* expression. Stage 6 to 10 embryos (3 to 5 hrs AEL) were subjected to a 1 hr heat shock treatment at 37°C and then allowed to develop until adulthood. *Mnn1* refers to strains harboring a UAS construct of this gene; expression is driven by either the nanos-GAL4 or tubulin-GAL4 drivers. Under heat shock conditions, organismal lethality of menin over-expressing lines (*nos*-*GAL4*/*Mnn1* and *tub*-*GAL4*/*Mnn1*) was attenuated when these strains were placed in an *Hsp70^-^* mutant background (*nos*-*GAL4*/*Mnn1*; *Hsp70* ^-^) or with a temperature sensitive *Hsf^-^* allele (*Hsf^4^*; *tub*-*GAL4*/*Mnn1*). Lethality of menin over-expressing lines was not attenuated by deletion mutants for a single *Hsp70* locus (*nos*-*GAL4*/*Mnn1*; *Hsp70B* ^-^). Percent lethality of wild-type (Oregon-R), parental control lines (*nos*-*GAL4*/+, *tub*-*GAL4*/+ and *Mnn1*/+) as well as homozygous *Hsf^4^* and *Hsp70* ^-^ mutants, and mutants homozygous for a single *Hsp70* locus (*Hsp70B^-^*) are shown for comparison. Stars denote significant differences between the indicated strains, p<0.05. Error bars represent SEM.

Lethality is observed in a high proportion of heat shock treated embryos over-expressing menin [16]. In these conditions, *Hsp70* and *Hsp23* are induced but are not down-regulated normally upon return to 25°C. In *Drosophila* larvae, the over-expression of *Hsp70* increases short-term thermotolerance to severe stress but decreases growth and survival to adulthood [24], [25]. Therefore, the lethality observed in conditions of *Mnn1* over-expression may be caused by the prolonged expression of *Hsp70* in these embryos. We asked whether a reduction in *Hsp70* gene copy number alters the lethality observed in conditions of *Mnn1* over-expression and heat shock. As shown in Fig. 4, the deletion of all *Hsp70* genes partially suppressed the lethality caused by *Mnn1* over-expression. Similar results were obtained in flies expressing a temperature-sensitive mutant of the heat shock factor inactivated at 37°C [*Hsf^4^*; [26]]. These results indicate that the lethality caused by *Mnn1* over-expression is due, at least in part, to the persistent expression of heat shock proteins and, in particular, of HSP70.

### Suppression of lethality associated with *Mnn1* mis-expression and stress by a dominant negative mutant of Chk2(mnk)

**Figure 5:**
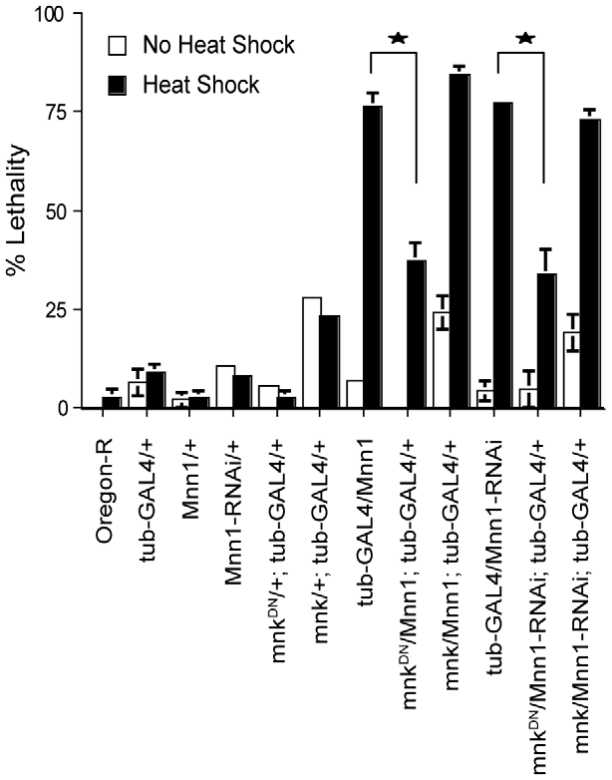
Menin interacts genetically with *Chk2(mnk)* in the heat shock response. Stage 6 to 10 embryos (3 to 5 hrs AEL) were subjected to a 1 hr heat shock treatment at 37°C and then allowed to develop until adulthood. *Mnn1* and *Mnn1*-*RNAi* refer to strains harboring a UAS construct for the over-expression and down-regulation of menin, respectively. *mnk* refers to a GUS construct of *Chk2(mnk)* for over-expression and *mnk^DN^* refers to a strain with a GUS construct for the expression of a dominant negative Chk2(mnk) kinase. The expression of all constructs is controlled by the tub-GAL4 driver. Wild-type (Oregon-R) and parental controls (*tub*-*GAL4*/+, *Mnn1*/+, *Mnn1*-*RNAi*/+) are shown for comparison. Organismal lethality observed in heat stressed *Drosophila* transgenic lines over-expressing (*tub-GAL4/Mnn1)* or down-regulating menin by RNA*i* (*tub-GAL4/Mnn1-RNAi*) was attenuated by co-expression of the dominant negative mutant of *Chk2*(*mnk)* (*mnk^DN^/Mnn1; tub-GAL4/+* and *mnk^DN^*/*Mnn1*-*RNAi*;*tub*-*GAL4*/+). No significant changes in lethality were observed in transgenic strains with mis-regulated *Mnn1* expression when wild-type *Chk2(mnk)* was over-expressed in the background (*mnk*/*Mnn1*; *tub*-*GAL4*/+ and *mnk*/*Mnn1*-*RNAi*; *tub*-*GAL4*/+). Stars denote significant differences between the indicated strains, p<0.05.

Heat shock treated embryos over-expressing or lacking menin are characterized by a high incidence of apoptosis and lethality [16]. In *Drosophila*, the Chk2*-*p53 pathway has been implicated in the control of apoptosis in response to gamma irradiation and DNA damage but the role of this pathway in response to other stresses has not been investigated [27], [28]. To determine the role of Chk2-p53 in the lethality caused by menin mis-expression, we first examined the effects of over-expressing *Chk2*(*mnk*) or a dominant negative mutant of this kinase in embryos subjected to a 1 hr heat shock treatment at 37°C. In embryos with a functional *Mnn1* gene, the over-expression of *Chk2*(*mnk*) increased lethality at the normal temperature and in response to heat shock (*mnk/+;tub-GAL4*/+ in Fig. 5). However, increasing the level of *Chk2*(*mnk*) did not enhance the lethality caused by *Mnn1* over-expression or loss-of-function. In contrast, the expression of the dominant negative mutant of *Chk2*(*mnk*) (designated mnk^DN^) suppressed partially the lethality observed in conditions of heat stress and menin mis-expression. This was true for embryos over-expressing menin (*UAS*-*Mnn1*) or a dsRNA for *Mnn1* (*UAS*-*Mnn1*-*RNAi*). Therefore, the lethality associated with *Mnn1* mis-expression and heat stress was dependent in part on *Chk2*(*mnk*) function.

Similar experiments were carried out to determine if the lack of p53 function alters the response of *Mnn1* null mutants to heat shock. However, unlike embryos expressing the dominant negative form of *Chk2*(*mnk*), *p53* null embryos were characterized by a significant degree of lethality in normal growth conditions. This lethality was also enhanced by heat shock, thus precluding the analysis of the role of p53 in the lethality caused by *Mnn1* mis-expression and stress (our unpublished results).

### Menin is a global regulator of the heat shock response

**Figure 6:**
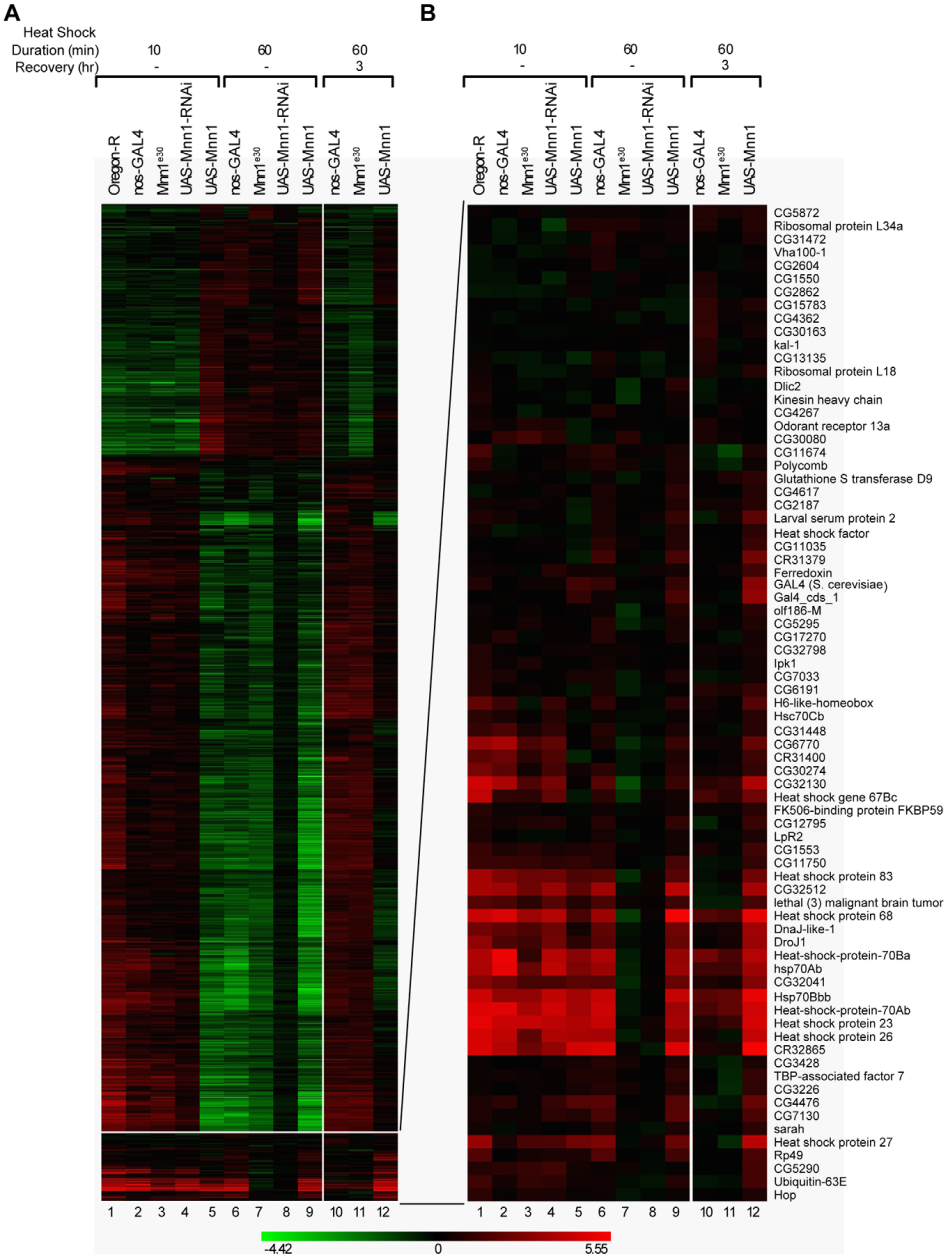
Menin is a global regulator of the heat shock response. **A**) Stage 6 to 10 embryos (3 to 5 hrs AEL) were subjected to either a 10 or 60 min heat shock treatment at 37°C or a 60 min heat shock plus 3 hr recovery at 25°C and RNA was isolated and analyzed using microarrays. Hierarchical clustering of differentially expressed genes is shown. A subcluster containing the heat shock genes is located at the bottom of the figure. The magnitude of differential expression for both the up-regulated (in red) and down-regulated (in green) genes is reflected by the intensity of color with the scale (in fold-change) shown below the figure. **B**) Enlargement of the subcluster containing the heat shock genes. Embryos deficient in menin protein (*Mnn1^e30^* and *UAS*-*Mnn1*-*RNAi*, lanes 7 and 8, respectively) are impaired in their transcriptional response to heat shock during prolonged treatments (60 min) but have normal patterns of heat shock gene expression at the 10 min time point (lanes 3 and 4). Embryos over-expressing menin (*UAS*-*Mnn1*, lane 12) are impaired in their ability to down-regulate the heat shock response during a 3 hr recovery at the normal temperature following a 60 min heat shock.

The results described above indicate that the genome instability caused by the absence of menin and heat stress cannot be explained by the aberrant expression of *Hsp70* (Fig. 3). Therefore, menin may act by mechanisms independent of gene expression and/or control the expression of multiple stress-responsive genes contributing to the maintenance of genome stability. To characterize the role of menin in the stress response, we performed a series of gene profiling studies of heat shock treated embryos over-expressing or lacking *Mnn1* function. In the first stage of this analysis, we identified more than 1000 genes with a significant difference in expression in response to heat shock or heat shock followed by recovery in at least one of the fly lines. Hierarchical clustering of the fold changes in the differentially expressed genes is shown in Fig. 6A (see also Supporting Information Dataset S1). A short (10 min) heat shock treatment at 37°C yielded overall patterns of gene expression that were similar in embryos of the different fly lines, with more genes being induced than repressed (lanes 1–4). The majority of genes differentially expressed at the 10 min time point showed modest levels of regulation of 1.5 to 2-fold. The exception was the menin over-expressing line (*UAS*-*Mnn1*), which displayed a pattern of gene expression resembling that of control embryos subjected to a longer 1 hr heat shock treatment (Fig. 6A, lanes 5 and 6).

Genes showing important fold-changes in induction include those encoding the major heat shock proteins. This group of genes is located in a single cluster shown in the lower part of Fig.6A and enlarged in Fig. 6B. A profound disruption in the expression of these genes was observed at the 60 min time point in the absence of menin, either as a result of gene deletion (*Mnn1^e30^*) or expression of a dsRNA (*UAS-Mnn1*-*RNAi*). In these conditions, none of these heat shock responsive genes were expressed after a 1 hr heat stress at 37°C, indicating that menin acted as a global regulator of heat shock protein genes (Fig. 6B, lanes 6–8). However, the induction of *Hsp* genes was normal after a 10 min heat shock treatment (Fig. 6B, lanes 1–4), suggesting that menin was not required for the initiation of the stress response but was critical for sustaining the expression of heat shock protein genes.

Differences in the profile of embryos over-expressing menin were also observed but several stress-responsive genes were induced normally in response to a 1 hr heat shock treatment at 37°C (Fig. 6B, lanes 6 and 9). However, striking differences were observed in menin over-expressing embryos during recovery from heat shock. In control embryos, the stress-inducible genes were down-regulated following a 3 hr recovery period at the normal temperature. In contrast, embryos over-expressing menin continued to express a sub-set of the heat shock protein genes including several of the *Hsp70* genes (Fig. 6B, lanes 10 and 12). Therefore, forced expression of menin impaired the down-regulation of major heat shock responsive genes such as those encoding HSP70. At the mRNA level, the effects of menin mis-expression were mostly limited to the regulation of stress responsive genes during heat shock and subsequently during recovery. In our conditions of heat shock (1 hr at 37°C), genes encoding known regulators of apoptosis, DNA repair or replication were not identified by gene profiling (Supporting Information Dataset S1). Thus, menin functioned as a global regulator of gene expression promoting sustained activation of heat shock responsive genes, a function required for the maintenance of genome integrity.

## Discussion

### Menin promotes genome stability in conditions of heat shock and hypoxia

In this study, we demonstrate that short, repeated but non-lethal conditions of heat shock or hypoxia increased genomic instability in *Mnn1* loss-of-function mutants. Unlike ionizing radiation or DNA crosslinking agents, heat shock is generally regarded as non-genotoxic. Nevertheless, the moderate conditions of heat shock or hypoxia used in these experiments resulted in levels of *mwh* LOH approaching those observed in p53 null flies subjected to ionizing radiation [27]. *Mnn1* deletion mutants are hypersensitive to ionizing radiation and DNA crosslinking agents such as nitrogen mustard and cisplatinum [12], [21]. Using a different test of LOH, the formation of tumors upon somatic loss of both alleles of the *lats* tumor suppressor gene, these authors observed similar increases in mutation frequencies (2–7 fold) when *Mnn1* loss-of-function mutants were subjected to gamma irradiation or treated with nitrogen mustard [12], [21]. These results highlight the importance of inducing an adequate stress response in the control of genome stability. They also suggest that a wide variety of conditions and factors, capable of inducing heat shock gene expression and the stress response, may affect genome stability, a conclusion with broad implications for our understanding of processes leading to oncogenesis. The increased lethality observed in p53 null embryos subjected to heat shock has not been reported before. This observation supports the notion that gene functions required for the maintenance of genome stability are also important for the response to stresses previously regarded as non-genotoxic (our unpublished observations).

Crosslinking agents generate small DNA lesions such as single base pair deletions. Consistent with the hypersensitivity displayed by *Mnn1* deletion mutants, the DNA lesions observed after treatment of *Mnn1* null mutants with nitrogen mustard or cisplatinum are small and consist primarily of single base pair deletions [12]. Whether or not the same type of DNA lesions are generated in response to non-genotoxic stresses, such as heat shock, is presently unknown. Characterization of these lesions would shed some light on the mechanism of action of menin. While similar DNA lesions may be observed in *Mnn1* loss-of-function mutants, it is also possible that several protein complexes controlling different biological processes are impaired in the absence of menin and proper expression of heat shock proteins. This question can be addressed with an adequate reporter assay, as described by others [12].

The expression of a dominant negative mutant of *Chk2(mnk)* suppressed partially the lethality observed in embryos over-expressing or lacking menin in response to heat stress (Fig. 5). In previous studies, we reported that menin mis-expression decreased the number of mitotic cells in heat shock treated embryos and resulted in a high proportion of cells arrested at metaphase during recovery [16]. These observations may be indicative of the activation of a chromosome or spindle checkpoint resulting from the effects of stress in *Mnn1* null embryos. Whether or not this phenotype reflects the accumulation of DNA damage and activation of *Chk2(mnk)* remains to be investigated. Independently of the mechanism(s) of action of menin, collectively, the results of our studies highlight the importance of the stress response in the maintenance of genome stability in *Drosophila*, a process dependent on the action of menin.

### Role of *Hsp70* in genome stability

Flies lacking all *Hsp70* genes were thermotolerant at 37°C but displayed a higher incidence of *mwh* loss-of-heterozygosity (Fig. 2B). Therefore, proper expression of *Hsp70* is important for genome stability in *Drosophila*. Interestingly, mice with a targeted deletion of *Hsp70.1* and *Hsp70.3*, the only genes encoding heat shock inducible forms of HSP70 in the mouse, are characterized by a high incidence of chromosomal abnormalities that is enhanced by heat stress [29]. Mouse embryo fibroblasts (MEF) from *Hsp70.1/Hsp70.3*-deficient animals display increased levels of chromosome end-to-end fusions and reduced viability in response to ionizing radiation (IR). *Hsp70.1/Hsp70.3* deficient MEF also have a higher rate of spontaneous transformation *in vitro*, indicating that proper expression of HSP70 is important for genome stability in flies and mammals.

While menin is a key regulator of *Hsp70* expression (Fig. 2A), *Hsp70* was apparently not the critical target of menin for the maintenance of genome integrity (Fig. 3). Indeed, the incidence of *mwh* LOH was enhanced when loss-of-function mutants of *Mnn1* were studied in the absence of functional *Hsp70* genes, suggesting that menin functions independently of *Hsp70* in our experimental conditions. This is perhaps not surprising considering that our repeated conditions of heat shock were limited to 20 min and that HSP70 expression was normal for the first 15 min of these treatments in *Mnn1* lack-of-function mutants (Fig. 2A). These results suggest that other or the combined action of multiple heat-shock responsive genes, controlled by menin, are required for the maintenance of genome stability in *Drosophila*. To address this possibility, we performed a series of gene profiling analyses to characterize the role of menin in the heat shock response. The results of these analyses (described below) indicated that menin functions as global regulator of heat shock gene expression. A more comprehensive study of heat shock responsive genes will be required to identify genes that play a more direct role in the control of genome stability in response to conditions such as heat shock.

Sustained and aberrant expression of *Hsp70* accounted in part for the lethality caused by menin over-expression and stress. This lethality was reduced significantly in *Drosophila* strains over-expressing *Mnn1* but lacking all copies of the *Hsp70* gene (Fig. 4). Since the absence of HSP70 decreases the capacity of the organism to cope with unfolded proteins, it is unlikely that the lethality observed in these experiments was simply due to a non-specific induction of the unfolded protein response caused by menin over-expression. A more likely interpretation is provided by the results of other investigators who reported that aberrant expression of *Hsp70* reduced growth and survival of *Drosophila* to adulthood [24], [25]. These results underscore the importance of a precise control of the stress response for survival of the organism.

### Menin is a global regulator of the heat shock response

We reported earlier that the expression of *Hsp70* and *Hsp23* is disrupted in loss-of-function mutants of the *Mnn1* gene [16]. In this study, we show that all heat shock protein genes are dependent on menin for proper expression in response to a 1 hr heat stress treatment (Fig. 6B). Therefore, menin functions as a global regulator of the heat shock response. However, we also note that over 1,000 genes were regulated by heat shock and expressed at similar levels in all fly lines analyzed in this study, suggesting that their regulation was largely menin-independent (Fig. 6A). This group of genes showed more modest changes in gene induction or repression (circa 1.5 to 2-fold difference) and may therefore be regulated by different mechanisms.

A role for menin in the activity of the Trithorax/MLL (Mixed Lineage Leukemia) family of histone methyltransferases is well established in mammals, suggesting a possible mechanism of action for menin in the control of heat shock gene expression in *Drosophila* [7], [8], [9]. As part of the TAC1 chromatin remodeling complex, Trithorax (Trx), the homolog of MLL1 in *Drosophila*, is required for proper expression of *Hsp70* in response to heat shock [30]. Menin and Trx co-immunoprecipitate in protein lysates of *Drosophila* S2 cells, suggesting that they are components of a protein complex conserved during evolution (our unpublished results). Whether or not the lack-of-function phenotype of *Mnn1* mutants can be accounted for by the disruption of the activity of Trx and TAC1 complex remains to be investigated.

Using a *Drosophila* line harboring a fusion of the *Hsp70* promoter and *LacZ* gene, we showed previously that menin regulates the activity of the *Hsp70* promoter [16]. In response to heat shock, *Drosophila* lines over-expressing menin (*UAS-Mnn1*) displayed increased β-galactosidase levels, which remained abnormally high upon return to the normal temperature. In contrast, the inhibition of menin expression by dsRNA (*UAS-Mnn1-RNAi*) reduced markedly the expression of the *Hsp70-LacZ* fusion gene, suggesting that menin is a direct regulator of the *Hsp70* promoter. However, one of the key observations of this study is that proper initiation of the heat shock response was independent of menin since a normal profile of heat shock gene expression was observed in *Mnn1* loss-of-function mutants subjected to a 10 min heat stress (Fig. 6). Forced expression of menin did not interfere with the induction of most heat shock responsive genes but impaired markedly the down-regulation of a subset of these genes during recovery at the normal temperature (Fig. 6B). Therefore, menin was dispensable for the initiation of the heat shock response but was required to sustain the expression of heat shock protein genes. The rapid repression of stress-responsive genes during recovery implies that the action of menin must be tightly controlled to prevent the unnecessary accumulation of heat shock proteins in the absence of stress.

The abrupt disappearance of *Hsp* transcripts and proteins in *Mnn1* loss-of-function mutants suggests that menin controls several aspects of the expression of heat shock mRNAs and proteins (Fig. 2A and 6B). Studies on the interaction of menin with signaling pathways controlling the expression of heat shock proteins are in progress.

In conclusion, we show that menin functions as a global regulator of the stress response promoting sustained expression of heat shock genes. This function is not only required for survival of the organism subjected to acute stress but is also critical for maintenance of genome integrity in conditions, such as heat shock, generally regarded as non-genotoxic. These results emphasize the importance of a precise control of the stress response and shed a new light on conditions promoting genome instability.

## Materials and Methods

### Fly stocks

Strains used in these studies were maintained on standard yeast-agar media in a 25°C incubator, and include Oregon-R, *tubulin (tub)-GAL4* (Bloomington No. 5138), *nanos* (*nos*)*-GAL4* (Bloomington No. 4442), *mwh^1^* (Bloomington No. 549), *Hsp70^-^* (also referred to as *Df(3R)Hsp70A*, *Df(3R)Hsp70B)* (Bloomington No. 8841), *Hsp70B^-^* (also referred to as *Df(3R)Hsp70B*) (Bloomington No. 8843), and *Hsf^4^* (Bloomington No. 5489). The mutant p53 line (*p53^1^*) and recombinant line *p53*, *mwh* were kindly provided by J. M. Abrams [27].

Transgenic lines *UAS-Mnn1* and *UAS-Mnn1-RNAi* were generated by P-element mediated transformation, as described before [16]. Deletion mutant strains *Mnn1^e30^* and *Mnn1^e173^* were generated by imprecise excision of a P-element and have been described previously [16]. In short, *Mnn1^e30^* harbors a deletion 2, 096-bp in size and *Mnn1^e173^* harbors a larger 4, 216-bp deletion. The transgenic fly strains *GUS-mnk^DN^* and *GUS-mnk* used in these experiments were kindly provided by M. Brodsky [31], [32].

### Loss-of-heterozygosity (LOH) assays

Flies heterozygous for a mutation in the *multiple wing hairs* (*mwh*) gene were used for all experiments on LOH [33], [34]. Standard genetic crosses were carried out to generate fly lines homozygous for either *Mnn1^e30^* or *Mnn1^e173^* in the heterozygous *mwh* background. Fly strains carrying one copy of *tub-GAL4* and heterozygous for *UAS-Mnn1* or *UAS-Mnn1-RNAi*, and/or *GUS-mnk^DN^* were also generated and compared to wild type (Oregon-R), and parental control lines heterozygous for *UAS-Mnn1*, *UAS-Mnn1-RNAi*, *GUS-mnk^DN^* or *tub-GAL4*. Five independent recombinant lines homozygous for *Hsp70^-^* and *mwh^1^* were generated and three strains homozygous for *Hsp70^-^* and heterozygous for *mwh^1^* were tested. Finally, nullizygous *Mnn1* strains (*Mnn1^e30^* or *Mnn1^e173^*), homozygous or heterozygous for *Hsp70^-^*, were also tested in LOH experiments.

For repeated, sub-lethal heat shock experiments, embryo progeny were collected in food vials and subjected to three 20 minutes heat shock pulses at 37°C in a water bath during embryogenesis (3 to 5 hr after egg laying [AEL]), first-instar larval stage (27 to 29 hr AEL), and second-instar larval stage (51 to 53 hr AEL). For hypoxia experiments, embryo progeny were dechorionated, placed into a sealed chamber, and subjected to hypoxia (10% O_2_) for a total of three 1 hour periods during embryogenesis (3 to 5 hr AEL), first-instar larval stage (27 to 29 hr AEL), and second-instar larval stage (51 to 53 hr AEL). In order to determine the effects of the short heat shock or low oxygen treatments on viability and LOH, progeny subjected to heat shock or hypoxia were allowed to recover until adulthood at 25°C. Lethality was calculated based on the expected number of mutant progeny relative to control siblings from the same cross. To assay LOH, wings were dissected, dehydrated in an ethanol series (50%, 70%, 80%, 90%, 100%) and mounted in 1∶1 methyl salicylate/permount (Fisher Scientific). All intervein wing hair cells were examined and cells with two or more hairs were scored as a *mwh^-^* phenotype. To eliminate any element of subjectivity when scoring the number of wing cells with multiple hairs, these counts were performed blind by two investigators. For each genotype and condition, thirty adult female wings were counted.

### Heat shock experiments

Stage 6 to 10 embryos (3 to 5 hr AEL) were collected in food vials, subjected to a 1 hour heat shock pulse at 37°C in a water bath, and allowed to recover until adulthood at 25°C in order to determine viability. Lethality calculation was based on the expected number of mutant progeny relative to the number of control siblings from the same cross. All embryos were heterozygous for the transposable elements indicated in Fig. 4. Three experimental crosses were carried out with a minimum of 100 progeny counted per cross. Western blotting analysis of HSP70 expression was carried out as previously described [16].

### Statistical analyses

One-way analysis of variance (ANOVA) and Student's unpaired *t*-test were used to determine the significance of differences between means. *P*<0.05 was considered statistically significant.

### RNA extraction, microarray experiments and data analysis

Total RNA was extracted from triplicate biological samples of each condition. RNA quality was evaluated using spectrophotometry and integrity was confirmed using gel electrophoresis of glyoxal-denatured samples. Samples were cleaned using MEGAclear kit (Ambion AM1908).

Total RNA samples were labeled using the ‘indirect’ method [35]. Superscript II reverse transcriptase (Invitrogen 18064014) was used to produce cDNA incorporated with aminoallyl-dUTP (Fermentas R0091). Reactive fluorescent dyes (Alexa647 or Alexa555; Invitrogen A32755) were conjugated to the individual samples. Two differently labeled samples, heat shocked and non heat shocked of the same genotype, were pooled and co-hybridized to the 14K long oligo array from the Canadian *Drosophila* Microarray Centre (www.flyarrays.com) according to previously described methods [36]. Images of the hybridized microarrays were obtained using a ScanArray 4000 scanner (Perkin-Elmer) and were quantified using QuantArray 3.0 software (Perkin-Elmer). Data were normalized using lowest sub-grid normalization [37] and Genetraffic Duo (Stratagene/Agilent) analysis software. Normalized data were exported and analyzed using the one-class test available in the Statistical Analysis of Microarrays (SAM) software package [38]. The false discovery rate of the one-class test was adjusted such that the expected number of false positive results was less than one. Gene lists generated in SAM were filtered to include only those genes that displayed at least a 1.5-fold change in abundance with respect to the non heat shock control sample, and whose coefficient of variance was less than 100%. Normalized log2 ratios were clustered using the Pearson algorithm [39] to reveal the relationship of the expression profiles across the varying conditions. The GEO accession number for the raw data of microarray analyses is GSE20497.

## Supporting Information

Table S1 A repeated heat shock or hypoxia regimen does not markedly increase organismal lethality. For heat shock experiments, progeny were subjected to a total of three 20 min heat shock treatments at 37°C during embryogenesis, first-instar and second-instar larval stages. For hypoxia experiments, progeny were subjected to hypoxia (10% O2) for a total of three 1 hr periods during embryogenesis, first-instar and second-instar larval stages. Progeny were allowed to recover until adulthood at 25°C to determine the effects of these treatments on viability. Lethality was calculated based on the expected number of mutant progeny relative to control siblings from the same cross. The heat shock or hypoxia regimen did not cause extensive lethality in any of the fly strains tested in these conditions. UAS-Mnn1 and UAS-Mnn1-RNAi are simply referred to as Mnn1 and Mnn1-RNAi, respectively.
(0.04 MB DOC)

Table S2 Hsp70- mutants survive heat shock at 37°C but not 39°C. The thermotolerance of 1-2 day old adult flies was tested using the protocol of Gong and Golic (23). Flies were pretreated with a 30 min heat shock at 35°C. This was followed immediately with a 40 min heat shock at either 39°C or 37°C. Following heat shock, flies were returned to 25°C to recover. The overnight survival was examined and the percent lethality is reported. Oregon-R flies survived heat shock at both temperatures; whereas Hsp70 null flies had a high lethality when exposed to heat shock at 39°C, as reported by Gong and Golic (23), but were able to tolerate the milder 37°C heat shock. For each genotype and condition, 75 flies were tested.
(0.03 MB DOC)

Dataset S1 Differentially Expressed Genes in Response to Heat Shock Treatments - Role of Mnn1 gene function. Expression values for samples and heat shock treatments described in Fig. 6 are provided. For each treatment and genotype, the ratio of the treated sample vs. the untreated sample for a given gene is shown as a fold change with down-regulated genes having a negative value.
(0.58 MB XLS)
