# Supplementary material for: Menin Links the Stress Response to Genome Stability in Drosophila melanogaster
Source: PLoS One. 2010 Nov 18;5(11):e14049. doi: 10.1371/journal.pone.0014049 (PMC2987805; doi:10.1371/journal.pone.0014049)
Supplement: Table S1 — A repeated heat shock or hypoxia regimen does not markedly increase organismal lethality. For heat shock experiments, progeny were subjected to a total of three 20 min heat shock treatments at 37°C during embryogenesis, first-instar and second-instar larval stages. For hypoxia experiments, progeny were subjected to hypoxia (10% O2) for a total of three 1 hr periods during embryogenesis, first-instar and second-instar larval stages. Progeny were allowed to recover until adulthood at 25°C to determine the effects of these treatments on viability. Lethality was calculated based on the expected number of mutant progeny relative to control siblings from the same cross. The heat shock or hypoxia regimen did not cause extensive lethality in any of the fly strains tested in these conditions. UAS-Mnn1 and UAS-Mnn1-RNAi are simply referred to as Mnn1 and Mnn1-RNAi, respectively. (0.04 MB DOC) [file pone.0014049.s001.doc]

**Supporting Information S1**

**Table S1. A repeated heat shock or hypoxia regimen does not markedly increase organismal lethality.**

|  | **% LETHALITY** | |
| --- | --- | --- |
| **GENOTYPES** | **HEAT SHOCK** | **HYPOXIA** |
| mwh/+ | 5 | 4 |
| Mnn1/+; mwh/+ | 3 | 3 |
| Mnn1-RNAi/+; mwh/+ | 7 | 10 |
| tub-GAL4/mwh | 0 | 0 |
| Mnn1/+; tub-GAL4/mwh | 6 | 8 |
| Mnn1-RNAi/+; tub-GAL4/mwh | 5 | 7 |
| Mnn1^e30^; mwh/+ | 7 | 10 |
| Mnn1^e173^; mwh/+ | 8 | 9 |
| Hsp70^-^, mwh/Hsp70^-^ (#1) | 6 | - |
| Hsp70^-^, mwh/Hsp70^-^ (#6) | 8 | - |
| Hsp70^-^, mwh/Hsp70^-^ (#8) | 5 | - |

For heat shock experiments, progeny were subjected to a total of three 20 min heat shock treatments at 37˚C during embryogenesis, first-instar and second-instar larval stages. For hypoxia experiments, progeny were subjected to hypoxia (10% O_2_) for a total of three 1 hr periods during embryogenesis, first-instar and second-instar larval stages. Progeny were allowed to recover until adulthood at 25˚C to determine the effects of these treatments on viability. Lethality was calculated based on the expected number of mutant progeny relative to control siblings from the same cross. The heat shock or hypoxia regimen did not cause extensive lethality in any of the fly strains tested in these conditions. UAS-*Mnn1* and UAS-*Mnn1*-RNAi are simply referred to as *Mnn1* and *Mnn1*-RNAi, respectively.
