# Supplementary material for: Menin Links the Stress Response to Genome Stability in Drosophila melanogaster
Source: PLoS One. 2010 Nov 18;5(11):e14049. doi: 10.1371/journal.pone.0014049 (PMC2987805; doi:10.1371/journal.pone.0014049)
Supplement: Table S2 — Hsp70- mutants survive heat shock at 37°C but not 39°C. The thermotolerance of 1-2 day old adult flies was tested using the protocol of Gong and Golic (23). Flies were pretreated with a 30 min heat shock at 35°C. This was followed immediately with a 40 min heat shock at either 39°C or 37°C. Following heat shock, flies were returned to 25°C to recover. The overnight survival was examined and the percent lethality is reported. Oregon-R flies survived heat shock at both temperatures; whereas Hsp70 null flies had a high lethality when exposed to heat shock at 39°C, as reported by Gong and Golic (23), but were able to tolerate the milder 37°C heat shock. For each genotype and condition, 75 flies were tested. (0.03 MB DOC) [file pone.0014049.s002.doc]

**Supporting Information S2**

**Table S2. *Hsp70*^-^ mutants survive heat shock at 37˚C but not 39˚C**

|  | **% LETHALITY** | |
| --- | --- | --- |
| **GENOTYPES** | **39˚C** | **37˚C** |
| Oregon-R | 5 | 0 |
| *Hsp70*^-^ | 71 | 3 |

The thermotolerance of 1-2 day old adult flies was tested using the protocol of Gong and Golic (23). Flies were pretreated with a 30 min heat shock at 35˚C. This was followed immediately with a 40 min heat shock at either 39˚C or 37˚C. Following heat shock, flies were returned to 25˚C to recover. The overnight survival was examined and the percent lethality is reported. Oregon-R flies survived heat shock at both temperatures; whereas *Hsp70* null flies had a high lethality when exposed to heat shock at 39˚C, as reported by Gong and Golic (23), but were able to tolerate the milder 37˚C heat shock. For each genotype and condition, 75 flies were tested.

Papaconstantinou et al
